# Protein Metalation by Medicinal Gold Compounds: Identification of the Main Features of the Metalation Process through ESI MS Experiments

**DOI:** 10.3390/molecules28135196

**Published:** 2023-07-04

**Authors:** Andrea Geri, Lara Massai, Luigi Messori

**Affiliations:** Department of Chemistry “Ugo Schiff”, University of Florence, Via della Lastruccia 3, 50019 Florence, Italy; andrea.geri@unifi.it

**Keywords:** gold compounds, ESI mass spectrometry, target proteins

## Abstract

Gold compounds form a new class of promising anticancer agents with innovative modes of action. It is generally believed that anticancer gold compounds, at variance with clinically established platinum drugs, preferentially target proteins rather than nucleic acids. The reactions of several gold compounds with a few model proteins have been systematically explored in recent years through ESI MS measurements to reveal adduct formation and identify the main features of those reactions. Here, we focus our attention on a group of five gold compounds of remarkable medicinal interest, i.e., Auranofin, Au(NHC)Cl, [Au(NHC)_2_]PF_6_, Aubipyc, and Auoxo6, and on their reactions with four different biomolecular targets, i.e., the proteins HEWL, hCA I, HSA and the C-terminal dodecapeptide of the enzyme thioredoxin reductase. Complete ESI MS data are available for those reactions due to previous experimental work conducted in our laboratory. From the comparative analysis of the ESI MS reaction profiles, some characteristic trends in the metallodrug-protein reactivity may be identified as detailed below. The main features are described and analyzed in this review. Overall, all these observations are broadly consistent with the concept that cytotoxic gold drugs preferentially target cancer cell proteins, with a remarkable selectivity for the cysteine and selenocysteine proteome. These interactions typically result in severe damage to cancer cell metabolism and profound alterations in the redox state, leading to eventual cancer cell death.

## 1. Introduction

Gold compounds form an attractive new class of experimental anticancer agents showing the remarkable ability to kill cancer cells with a good degree of selectivity [1,2,3]. This makes them excellent drug candidates suitable for further biological and pharmacological investigation. Indeed, in recent years, a large variety of gold compounds in the oxidation states +3 and +1 have been prepared and evaluated for in vitro anticancer properties with rather encouraging results [4,5,6,7]. These experimental medicinal compounds include either gold(I) or gold(III) complexes [8]. A number of these compounds that manifested highly favorable chemical and biological features have been selected for more systematic and advanced biological studies in our laboratory. Mechanistic studies point out that medicinal gold compounds react preferentially with protein molecules rather than with nucleic acids; this makes their mechanism of action innovative and very different from that of clinically established platinum drugs [9,10].

This latter observation has triggered a lot of attention toward the analysis of protein metalation sustained by gold-based drugs [11,12].

Protein metalation is the process through which a metal-based drug modifies a selected protein and alters its function; to gain deeper mechanistic insight, it is important to define the general features of protein metalation by gold compounds [13,14]. The study of protein metalation by metal-based drugs has attracted a conspicuous interest in recent years in the scientific community; a lot of detailed information on the protein metalation processes and on the resulting metal-protein adducts has been obtained through combined implementation of X-ray crystallography and ESI MS methods [15,16,17,18]. Specifically, ESI MS has turned out to be an excellent tool for characterizing the protein interactions of metallodrugs that occur in the solution. Indeed, the ESI MS experiment is easy, rapid, cheap, and very informative [19,20].

**Figure 1:**
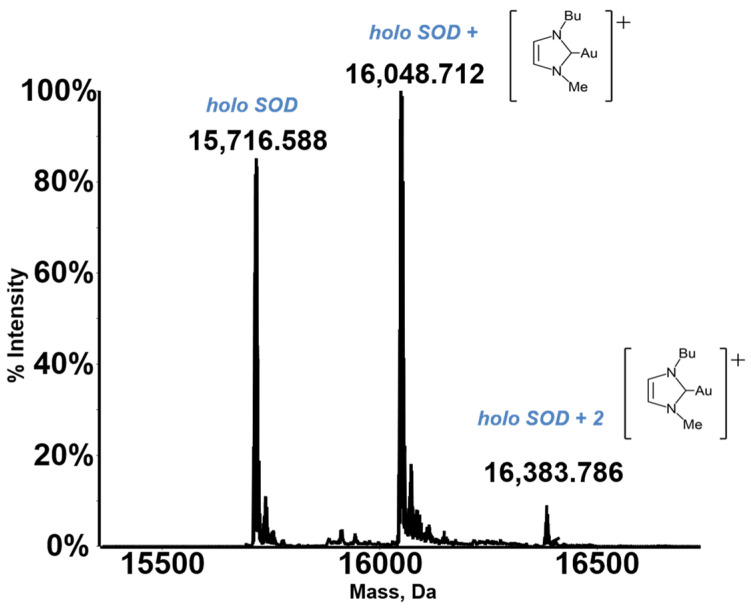
An exemplary ESI MS spectrum. Deconvoluted mass spectra of SOD 10^−7^ M in 2 mM ammonium acetate solution at pH 6.8 incubated at 37 °C for 24 h with Au(NHC)Cl in a 1:3 protein-to-gold ratio [21]. Adapted with permission from Ref. [21]. 2021 Elsevier.

A characteristic deconvoluted ESI MS spectrum for a metallodrug-protein adduct is reported in Figure 1.

Notably, the resulting deconvoluted spectrum shows the peak of the native protein and some additional peaks, with a greater molecular mass, that are quite easily assigned to the formed metallodrug-protein adducts. Thus, the ESI MS experiment may provide straightforward information on the extent of adduct formation compared to the free protein, the stoichiometry and the distribution of the resulting adducts, and the nature of the protein-bound metallic fragment. In addition, through ESI MS time course measurements, it is possible to gain detailed insight into the rate of adduct formation.

## 2. The Gold Compounds Chosen for the Interaction Studies

The present short review is focused on a group of five gold compounds of particular interest in medicinal chemistry. This group includes the following compounds: Auranofin, Au(NHC)Cl, [Au(NHC)_2_]PF_6_, Aubipyc, and Auoxo6.

During the past years, three of the above-mentioned gold complexes, i.e., Auranofin, Auoxo6, and Aubipyc, were evaluated in vitro at Oncotest, (Freiburg, Germany) against a panel of 36 different human tumor cell lines [22] and all of them manifested interesting antiproliferative properties. Moreover, the FDA approved anti arthritic drug, Auranofin, has been enrolled in three distinct clinical trials for cancer treatment (ClinicalTrials.gov Identifier: NCT01419691, NCT01747798, NCT01737502).

In addition, these gold compounds were evaluated against a conventional tumor cell line, the A2780 ovarian cancer cell line; all of them produce potent cytotoxic effects with IC_50_ values typically falling in the low micromolar range (see Table 1), and in some cases the efficacy is greater than that of cisplatin.

**Figure 2:**
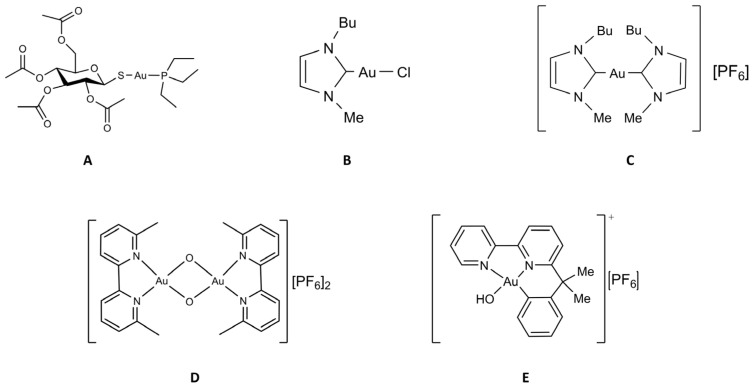
Chemical structure of the selected gold compounds. *(***A**) Auranofin; *(***B**) Au(NHC)Cl; (**C**) [Au(NHC)_2_]PF_6_; *(***D**) Auoxo6; *(***E**) Aubipyc.

Some more information on these gold compounds is given below (see Figure 2).

### 2.1. Auranofin

Auranofin (AF hereafter) is a gold (I)-containing compound that was approved by the United States Food and Drug Administration in 1985 as a primary treatment against rheumatoid arthritis [27,28]. AF consists of a mononuclear gold(I) center linearly coordinated to a triethylphosphine molecule and to 1-thio-β-D-glucose-2,3,4,6-tetraacetate. This drug behaves as a prodrug, undergoing irreversible oxidation of the thioglucose tetraacetate accompanied by hydrolysis, leading to progressive deacetylation. This results in the generation of two main active forms: a triethylphosphinenegold (I) cation, and a gold(I) thioglucose species, with a variable number of acetyl groups [22,23]. Owing to its chemical features and its encouraging antiproliferative actions and acceptable toxicity profile, AF is now being repurposed for cancer treatment [29,30,31]. It is known that AF interacts very weakly with DNA while manifesting a remarkable affinity and selectivity for proteins bearing free cysteines and selenocysteines [32,33].

### 2.2. The Two Gold Carbenes, Au(NHC)Cl and [Au(NHC)_2_]PF_6_

N-Heterocyclic carbenes (NHCs) are very fascinating gold(I) ligands as they possess donor properties similar or superior to phosphines, thus providing very stable gold(I) complexes [34,35]. In recent years we have synthesized, chemically characterized and biologically tested two gold(I) carbene complexes (Figure 2) that are structurally related [24]. In fact, in both complexes, the gold(I) center linearly coordinates a 1-butyl-3-methylimidazole-2-ylidene ligand and, as second ligand, a chloride (Au(NHC)Cl) or another identical N-heterocyclic carbene ([Au(NHC)_2_]PF_6_). Due to the presence of a relatively weak ligand (i.e., the chloride, that is prone to be released by the metal atom) in the mono-carbene complex, the two compounds are deeply distinct from a chemical point of view and in terms of the overall charge: Au(NHC)Cl is neutral, less stable, and more reactive while [Au(NHC)_2_]PF_6_ is mono-cationic, highly stable and poorly reactive. Both compounds manifested promising and potent anticancer actions in a variety of in vitro models [36,37].

### 2.3. Auoxo6

Auoxo6, ((6,6′-dimethyl-2,2′-bipyridine)_2_Au_2_(µ-O)_2_)(PF_6_)_2_), is a dioxo-bridged dinuclear gold(III) complex that was previously reported to exhibit significant and favorable anticancer effects in vitro toward a large number of human cancer cell lines [22].

The coordination of the two gold(III) centers is roughly square planar, the system containing the so called Au_2_O_2_ “diamond core”; the tetracoordination of both gold(III) centers is completed by two nitrogens belonging to the aromatic rings of the bipyridine ligands [38,39]. The compound reveals an acceptable stability under physiological conditions. In any case, the key processes characterizing Auoxo6’s reactivity appear to be: (i) gold(III) reduction, (ii) dioxo bridge disruption, (iii) coordinative gold(I) binding to proteins, and (iv) concomitant release of the N-heterocyclic ligand; yet, the precise modes/mechanisms of action remain partially unknown [16,34,35].

### 2.4. Aubipyc

Aubipyc is a gold(III) cyclometalated derivative of 6-(1,1-dimethylbenzyl)-2,2′-bipyridine, characterized by a square planar arrangement of the gold(III) center [26]. One of the gold donors is a carbon atom while the other three substituents are two nitrogens from the bipyridyl ligand and a hydroxide group. The presence of a direct gold–carbon bond greatly stabilizes the gold(III) center and limits its tendency to undergo reduction to gold(I). This compound was shown to possess considerable antiproliferative properties against a panel of 12 cancer cell lines and to interfere with carbohydrate metabolism [26,40,41].

Notably, this short review re-evaluates the ESI MS interaction studies between the above gold compounds and four representative model protein and peptides. In particular, we directed out attention to HEWL, hCA 1, HSA, and the C-terminal peptide of the selenoenzyme Thioredoxin reductase, that are highly representative and important biomolecular systems and good models for the real targets [42].

## 3. Adduct Formation between Medicinal Gold Compounds and Model Proteins Disclosed by ESI MS Measurements

ESI-MS is a valuable tool for the analysis of the interactions between metal complexes and biomolecules in solution [43].

Systematic ESI MS studies have now been carried out to characterize the reactions of several gold compounds of medicinal interest with a variety of representative model proteins and peptides. In particular, we noticed that complete sets of experimental data are already available for the reactions of the above five gold compounds with the four mentioned protein and peptides, i.e., HEWL, hCA 1, has, and the C-terminal dodecapeptide of thioredoxin reductase. HEWL and hCA 1 were chosen for their characteristics of good ionizability and small size, whereas the dodecapeptide was selected because it derives from a protein (TrxR) that is a well-known target for antitumor gold drugs; the HSA is the most abundant protein in the plasma, and the possible interaction with this biomolecule is a crucial event for a lot of drugs.

Since the interactions between gold compounds and DNA seem to be weaker and less selective than the interactions between gold compounds and proteins, these latter were the preferred for the analysis.

Here, all these experimental results are synoptically reported and comparatively analyzed in order to identify the main trends of the protein metalation processes. The most important results are recapitulated below.

### 3.1. Lysozyme (HEWL)

**Figure 3:**
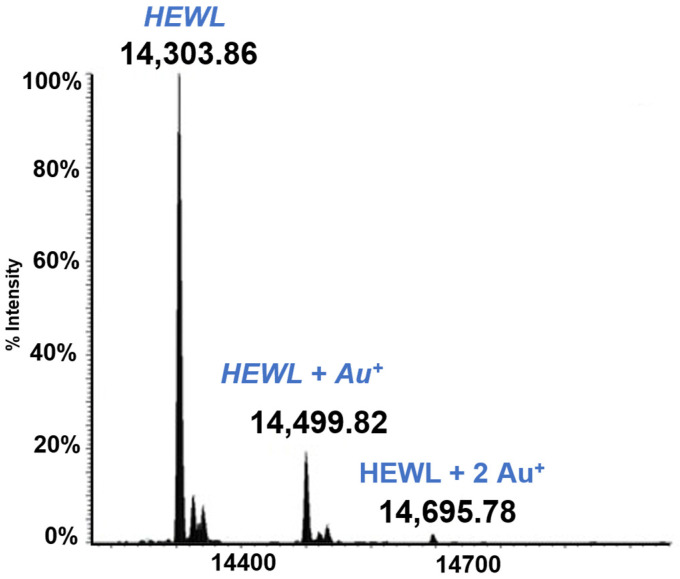
LTQ Orbitrap electrospray ionization (ESI) mass spectra of Lysozyme (HEWL) 10^−5^ M in 25 mM tetramethylammonium acetate (TMeAmAc) buffer, pH 7.4, and incubated at 37 °C for 72 h with Auoxo6 in a 1:3 protein-to-gold ratio [16].

Lysozyme is a small globular protein (14KDa) that has been extensively used for protein metalation studies. Those studies revealed that the main metalation site of this protein is the solvent exposed His 15. Complete experiments have now been carried out where HEWL was reacted individually with the five gold compounds of the above panel. From those studies it clearly emerges that HEWL is not very prone to form adducts with these gold compounds. Only in the case of Auoxo6 was an adduct clearly formed (see Figure 3). This adduct corresponds to the binding of a Au+ ion to the protein; this finding supports the view that adduct formation takes place upon AuOxo6 reduction and ligand release [17]. Crystallographic results were nicely consistent with this finding and revealed that gold binding occurs at the level of His 15 [44]. In all the other cases no adduct formation was observed under the applied solution conditions.

### 3.2. hCA I

**Figure 4:**
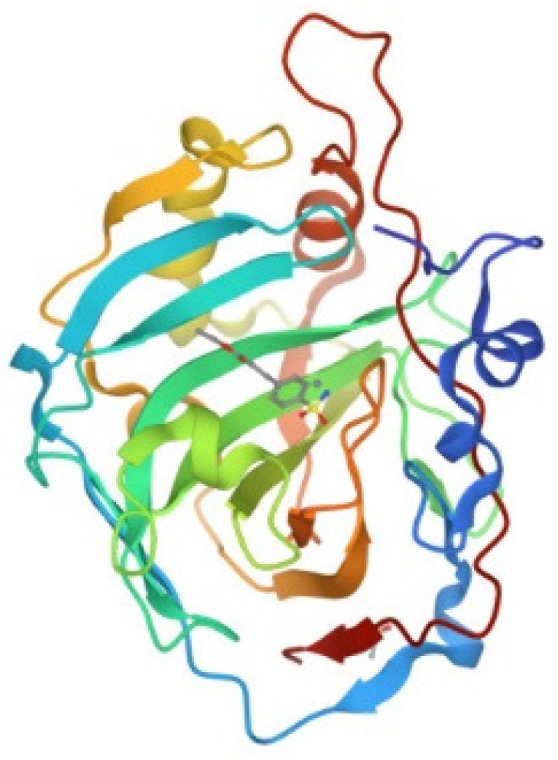
Ribbon representation of the overall structure of human carbonic anhydrase I (PDB entry 2NN7).

**Figure 5:**
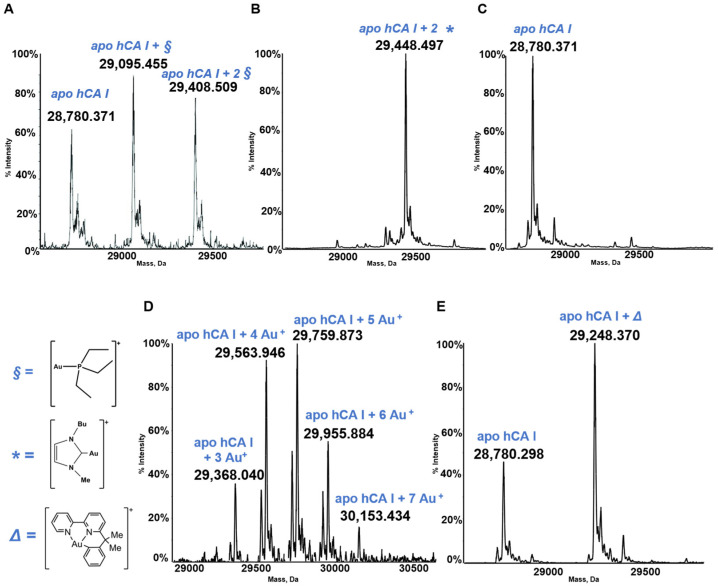
Deconvoluted electrospray ionization quadrupole time-of-flight (ESI-Q-TOF) spectra of human carbonic anhydrase I (hCA I) 7 × 10^−7^ M in 2 mM of ammonium acetate solution at pH 6.8 and incubated at 37 °C for 24 h with (**A**) AF; (**B**) Au(NHC)Cl; (**C**) [Au(NHC)_2_]PF_6_; (**D**) Auoxo6 and (**E**) Aubipyc solution in dimethyl sulfoxide (DMSO) in a 1:0.9 protein-to-gold ratio [25,40,41]. The symbols §, *, ∆ indicate the metal fragments that bind the protein.

Human carbonic anhydrase I, hCA I, is a globular protein of greater mass than lysozyme (ca 29KDa) that bears a solvent accessible Cys residue with a free sulfhydryl group not involved into a disulfide bond, i.e., Cys213. hCA I is a zinc enzyme catalyzing very efficiently carbon dioxide hydration (Figure 4). The reactions of this protein with panel gold compounds were systematically analyzed by ESI MS. Notably, the reaction led, in all cases, to adduct formation with the only exception of the gold dicarbene complex. Most likely, adduct formation occurs at the level of the free cysteine. Well resolved ESI MS spectra were obtained for the various adducts, as shown in Figure 5. 

Well defined adducts are formed upon hCA I’s reaction with both AF and Au(NHC)Cl (Figure 5). The protein bound metallic fragment corresponds to AuPEt_3_^+^ in the case of AF and to [Au(NHC)]^+^ in the case of the monocarbene gold complex [21,32]. As stated above, no adduct originates from the reaction of hCA I with the biscarbene complex. Adduct formation is clearly detected upon reacting hCA 1 with the gold(III) compounds Auoxo6 and Aubipyc. Au^+^ and [Au(III)(bipydmb-H)]^2+^ are the resulting protein bound fragments. These observations suggest that Auoxo6 undergoes reduction and is able to bind different amino acid residues (cys, his), as well witnessed by different techniques, such as spectrophotometric measurements and crystallographic data [44,45]; the gold(III) oxidation state is conserved in the reaction of Aubipyc with the same protein owing to the stabilization effects produced by the ligand [45].

### 3.3. HSA

**Figure 6:**
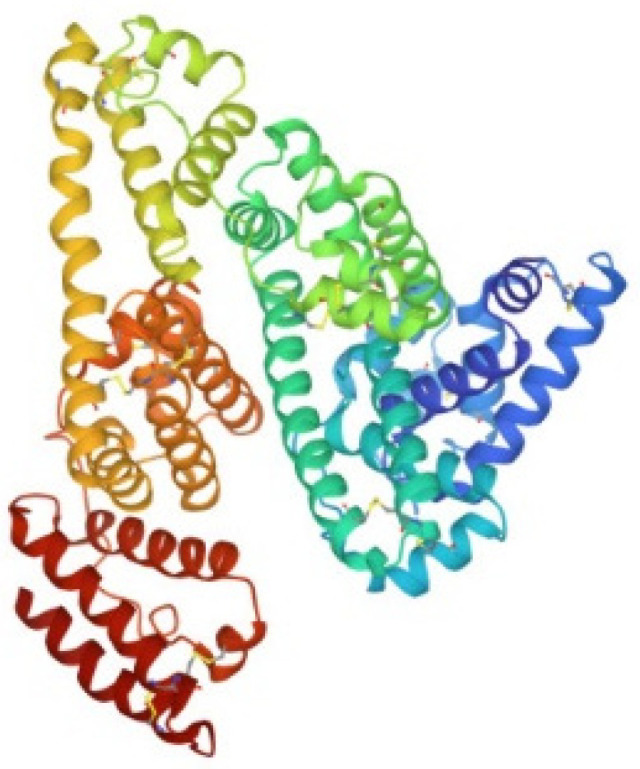
Ribbon representation of the overall structure of human serum albumin (PDB entry 1AO6).

**Figure 7:**
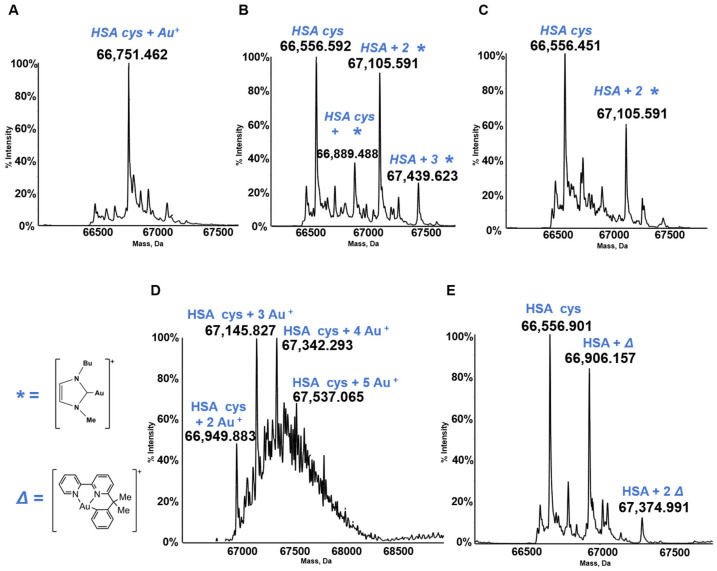
Deconvoluted electrospray ionization quadrupole time-of-flight (ESI-Q-TOF) spectra of human serum albumin (HSA) 5 × 10^−7^ M in 2 mM of ammonium acetate solution at pH 6.8 and incubated at 37 °C for 24 h with (**A**) AF; (**B**) Au(NHC)Cl; (**C**) [Au(NHC)_2_]PF_6_; (**D**) Auoxo6 and (**E**) Aubipyc solution in dimethyl sulfoxide (DMSO) in a 1:3:5 protein-to-gold-to-DTT ratio [40,41,42]. The symbols *, ∆ indicate the metal fragments that bind the protein.

HSA is the most abundant serum protein. HSA is a relatively big globular protein (around 65 KDa) that comprises three distinct domains (see Figure 6). In spite of its large size, reasonably well resolved ESI MS spectra may be taken for HSA and for its metallodrug derivatives. Accordingly, HSA has been reacted individually with the five panel gold compounds and the corresponding ESI MS deconvoluted spectra were recorded after 24 h (see Figure 7). Notably, adducts are formed with all gold compounds although in different abundances. The nature of the protein bound gold fragments is specified in the spectra [21,45,46].

### 3.4. The TrxR Dodecapeptide

**Figure 8:**
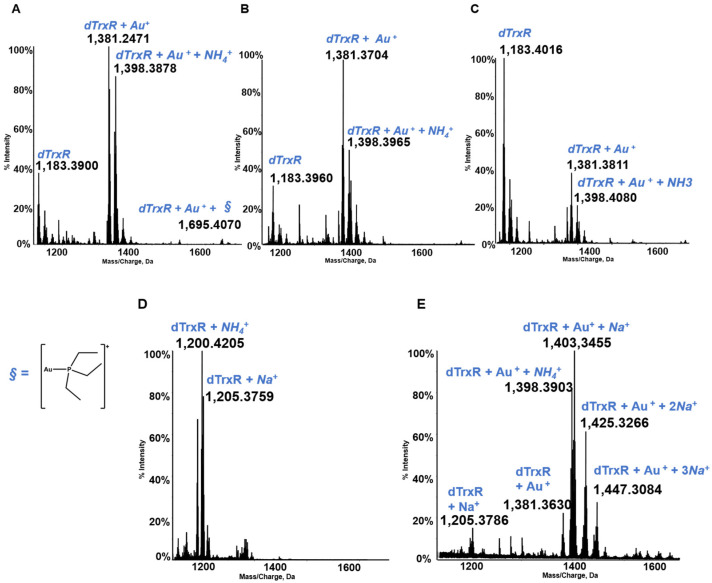
Multicharged electrospray ionization quadrupole time-of-flight (ESI-Q-TOF) spectra of the dodecapeptide 5 × 10^−7^ M in 2 mM of ammonium acetate solution at pH 6.8 and incubated at 37 °C for 2 h with (**A**) AF (unpublished data, from laboratory archive); (**B**) Au(NHC)Cl; (**C**) [Au(NHC)_2_]PF_6_; (**D**) Auoxo6; and (**E**) Aubipyc solution [40,41]. The symbol § indicate the metal fragments that bind the protein.

Thioredoxin reductase is an important selenoenzyme, crucially implicated in the regulation of the intracellular redox state, that is present both in the cytosol and in the mitochondria of cancer and healthy cells [47,48]. Thioredoxin reductase is believed to represent the main target protein for medicinal gold compounds in view of the large affinity of the gold(I) center for the active site selenol-thiol group of the enzyme [49,50,51]. Unfortunately, the protein is too big and does not afford well-resolved ESI MS spectra, so in several of our papers the reaction of gold compounds with the active site of TrxR containing the selenol-thiol moiety was evaluated through the interaction, not with the full TrxR proteins but with the C terminal dodecapeptide of thioredoxin reductase. In view of the large affinity of the gold center for the selenol group, we thought that the occurring interactions might be well representative of the interactions taking place between the various gold compound and the entire protein [52]. The dodecapeptide was, thus, reacted with panel gold compounds, and the resulting ESI MS spectra were recorded (see Figure 8). All these reactions were conducted under reducing conditions in order to cleave the Se–S bond. These conditions invariably led to the formation of adducts containing one or more gold(I) ions bound to the dodecapeptide. The obtained ESI MS spectra and the assignment of the adducts obtained in the various cases are shown in Figure 8; it is evident that owing to the presence of a strongly reducing environment, adducts only contain one or more gold(+) ions. The original gold ligands are invariably lost [21,45].

## 4. Discussion

The comparative analysis of the above ESI MS data permits the identification of some general trends in the reactivity of panel gold compounds with model proteins. The main features of those interactions emerging from the above analysis are summarized below:

i. Gold compounds behave as prodrugs and must undergo chemical transformation in order to react with proteins.

Gold drugs typically behave as prodrugs, i.e., they need activation in order to react with proteins; indeed, ESI MS results show that the analyzed gold drugs undergo transformation and generate metallic fragments able to bind proteins. In the case of gold(I) drugs these fragments typically correspond to the loss of one of the two gold ligands that are present in the original complexes, i.e., the weaker one. The resulting metallic fragment may associate to proteins through formation of a strong gold-to-protein coordination bond. This clearly happens for AF and Au(NHC)Cl. The more labile ligand detaches so that the AuPEt_3_^+^ fragment in the case of AF and the Au(NHC)^+^ fragment in the case of Au(NHC)Cl will bind the protein. Gold(III) drugs also behave as prodrugs but their activation process may be more complex and may require concomitant redox chemistry as mentioned below. This is the case of Auoxo6 in its reactions with HEWL, hCAI, HSA, and the dodecapeptide of TrxR. In contrast the activation process in the case of the organometallic gold(III) complex Aubipyc mainly involves the release of the weak ligand with retention of the stronger terdentate ligand and of the oxidation state +3 of the gold center.

ii. The reactivity of gold compounds, its extent and selectivity are tightly controlled by the nature of the leaving group.

This aspect is strictly connected to the previous one. The reactions of AF, Au(NHC)Cl, and [Au(NHC)_2_]PF_6_ with hCA I are particularly instructive in this respect. Indeed, the chloride ligand in Au(NHC)Cl is a good leaving group so that adduct formation is relatively easy. The thiosugar ligand in AF is a somewhat stronger ligand but its replacement is favored over the replacement of the phosphine ligand. Thus, protein metalation takes place and involves protein binding of the AuPEt_3_^+^ fragment. In contrast, the fact that NHC is a very tight and stable ligand, thanks also to its donor properties [53] and a bad leaving group, well accounts for the inability of [Au(NHC)_2_]PF_6_ to form adducts with hCA I. The presence of tight ligands on the gold center may result in a reduced and more selective reactivity [54,55]. Within the panel gold(III) complexes Aubipyc behaves as a classical prodrug with release of the weak ligand; in the case of Auoxo6 the reaction is instead governed by the reduction process that releases free gold(I) ions. The latter are able to bind the protein targets.

iii. Upon reaction gold containing molecular fragments associate tightly to proteins through formation of strong coordinative bonds

Upon reaction, the gold center is able to coordinate relatively strongly to exposed protein residues. The gold center may bind to the protein as a gold containing molecular fragment or as a free ion. Metal coordination to protein sidechains results in the formation of relatively stable adducts. Typically, these adducts remain stable in the ESI MS ionization process and thus may be directly detected.

iv. Gold binding is highly selective for free cysteine (and selenocysteine) residues.

Gold compounds manifest a pronounced preference for free thiol (or even the more rare selenol) groups and a scarce tendency to coordinate to other protein side chains [56]. This is the reason why gold(I) compounds are commonly unable to form adducts with lysozyme, a protein that is devoid of solvent accessible free thiols. In contrast, gold(I) compounds generally form large amounts of adducts with the protein hCA 1 bearing a free thiol group. Notably, it was shown that blocking the only free cysteine in hCA1 results in a strong reduction in adduct formation. Indeed, the residual binding affinity for other protein sidechains is relatively small.

v. Gold binding to specific protein residues may result in proteins’ loss of function.

In the case the cysteine or selenocysteine residue undergoing metalation plays a relevant functional role in the protein, its metalation will definitely result in protein’s loss of function. The C terminal dodecapeptide of thioredoxin reductase manifests a large reactivity with gold compounds; at the same time, this dodecapeptide is essential for the catalytic process of thioredoxin reductase. It follows that metalation of this selenol/thiol motif will result in potent enzyme inhibition as it was extensively documented for several gold compounds [34,47,48].

vi. In the case of gold(III) compounds, protein binding is often preceded by the reduction of gold(III) to gold(I)

As stated above, adduct formation in the case of the gold(III) complexes may be associated and dependent on a redox process. This observation applies well to gold(III) compounds bearing a high oxidizing character. Reduction of gold(III) to gold(I) typically leads to the release of the original metal ligands that manifest a large affinity for the gold(III) center and a significantly lower affinity for the gold(I) center. Thus, the resulting protein adducts often contain one or more protein bound gold(I) ions that have lost its(their) original ligands; only in cases where the metal environment is able to stabilize the gold(III) center, as it happens for Aubipyc, we find adducts with gold in the oxidation state +3 and retention of the ligands.

## 5. Conclusions

Gold compounds form an innovative class of cytotoxic and anticancer drugs. The biological profile of gold drugs is profoundly distinct from that of clinically established anticancer Pt drugs. Differences in the respective biological profiles are traced back to the occurrence of substantial differences in their modes of action. Indeed, while Pt drugs target preferentially genomic DNA and nucleic acids, gold drugs exert their actions mostly through binding to protein targets.

The ability to interact strongly with different proteins is dependent on several factors, such as the kinetic lability of the ligand, i.e., the chloride of the Au(NHC)Cl or the hydroxyl group of the Aubipyc, or the redox properties of complexes, i.e., the Auoxo6 compound. Furthermore, the thousands of proteins present in the cell are related to many different cellular networks and the inhibition of each of them could [8] impair more than one biological pathway. So, the resulting cytotoxicity of gold compounds is given by the contribution of several factors; first of all it is determined by the nature of the various targets and by the chemical structure of the complexes.

With this review, we attempted to summarize the types of reactivity of medicinal gold compounds with proteins according to the differences among the selected panel of the complexes, such as oxidation number and nature of the ligands. We have considered, in detail, a number of studies where the reactions of five characteristic medicinal gold compounds with four model proteins are comparatively investigated through ESI MS measurements. Characteristic trends in reactivity have emerged from the detailed analysis of the ESI MS data. Notably, the observed patterns of reactivity critically depend on the chemical nature of the gold compound and the structural characteristic of the interacting protein. In any case, the prodrug nature of the various gold compounds and their high selectivity for solvent-exposed free thiols (selenols) have been unambiguously demonstrated.

## Figures and Tables

**Table 1:** The cytotoxic activity of the selected gold complexes towards A2780 human ovarian carcinoma cell lines over 72 h drug treatment.

Gold Compounds	A2780 IC_50_ (µM) ± SD	Ref.
Auranofin	0.50 ± 0.39	[23]
Au(NHC)Cl	1.98 ± 0.17	[24]
[Au(NHC)_2_]PF_6_	0.10 ± 0.02	[24]
Auoxo6	1.79 ± 0.17	[25]
Aubipyc	3.30 ± 1.40	[26]

## Data Availability

Data is contained within the article. The data presented in this study was obtained from the references indicates in the Figure 1, Figure 3, Figure 5, Figure 7 and Figure 8 and in the Table 1.

## Sample Availability

Samples of the metal compound and the proteins are available from the authors.
